# Supplementary material for: Protein Intake, Fatigue and Quality of Life in Stable Outpatient Kidney Transplant Recipients
Source: Nutrients. 2020 Aug 14;12(8):2451. doi: 10.3390/nu12082451 (PMC7469059; doi:10.3390/nu12082451)
Supplement: Supplementary file 1 [file nutrients-12-02451-s001.pdf]

Table S1. Final cumulative model of multinomial regression analyses of protein intake (per 10g/d increment) with fatigue in KTR

	No Fatigue		Moderate Fatigue		Severe Fatigue	
CIS	<20		20-34		≥ 35	
	OR (95% CI)	P-value	OR (95% CI)	P-value	OR (95% CI)	P-value
Protein intake	Reference	(-)	0.89 (0.80 – 1.00)	0.06	0.80 (0.71 – 0.91)	< 0.001
Age	Reference	(-)	1.00 (0.98 – 1.01)	0.75	1.00 (0.98 – 1.02)	0.94
Sex	Reference	(-)	1.19 (0.71 – 1.98)	0.51	0.84 (0. 49 – 1.44)	0.53
BMI	Reference	(-)	1.05 (1.0 – 1.11)	0.05	1.10 (1.04 – 1.17)	<0.001
Diabetes	Reference	(-)	1.56 (0.91 – 2.66)	0.10	2.25 (1.31 – 3.88)	0.003
Pre-emptive Tx	Reference	(-)	0.65 (0.40 – 1.05)	0.08	0.56 (0.34 – 0.94)	0.03
eGFR	Reference	(-)	1.00 (0.98 – 1.01)	0.46	0.99 (0.98 – 1.01)	0.29
Primary kidney disease						
- Glomerulonephritis	Reference	(-)	1.12 (0.57 – 2.22)	0.74	2.46 (1.20 – 5.01)	0.01
- Interstitial nephritis	Reference	(-)	0.81 (0.35 – 1.87)	0.63	0.82 (0.33 – 2.04)	0.67
- Cystic Kidney Disease	Reference	(-)	0.69 (0.35 – 1.36)	0.28	1.15 (0.55 – 2.38)	0.72
- Other congenital and hereditary kidney disease	Reference	(-)	0.91 (0.30 – 2.83)	0.87	1.63 (0.51 – 5.23)	0.41
- Renovascular disease	Reference	(-)	1.06 (0.51 – 2.21)	0.88	0.71 (0.30 – 1.68)	0.44

- Diabetes	Reference	(-)	0.67 (0.19 – 2.39)	0.54	1.51 (0.45 – 5.10)	0.51
- Other multisystem diseases	Reference	(-)	1.43 (0.45 – 4.51)	0.55	1.35 (0.36 – 5.01)	0.66
- Other	Reference	(-)	0.61 (0.13 – 2.94)	0.54	1.34 (0.30 – 5.98)	0.70
Proteinuria	Reference	(-)	0.51 (0.25 – 1.01)	0.05	1.04 (0.54 – 2.02)	0.90
Time since Tx	Reference	(-)	1.02 (0.99 – 1.06)	0.18	1.03 (1.00 – 1.07)	0.08
Use of cyclosporin	Reference	(-)	0.83 (0.44 – 1.59)	0.58	1.31 (0.70 – 2.48)	0.40
Use of mycophenolic acid	Reference	(-)	1.25 (0.71 – 2.22)	0.44	0.87 (0.48 – 1.56)	0.63
History of combined organ Tx	Reference	(-)	13.6 (1.37 – 135)	0.03	12.6 (1.30 – 123)	0.03
SBP	Reference	(-)	1.00 (0.98 – 1.01)	0.73	0.99 (0.98 – 1.01)	0.36
Cholesterol	Reference	(-)	0.91 (0.72 – 1.16)	0.46	1.03 (0.81 – 1.31)	0.82
Statin use	Reference	(-)	1.15 (0.72 – 1.81)	0.56	0.74 (0.46 – 1.19)	0.21
Hemoglobin	Reference	(-)	1.06 (0.84 – 1.34)	0.63	0.97 (0.76 – 1.24)	0.80
Ferritin	Reference	(-)	1.00 (0.99 – 1.00)	0.70	1.00 (1.00 – 1.00)	0.57
Vitamin B12	Reference	(-)	1.00 (0.99 – 1.00)	0.57	1.00 (1.00 – 1.00)	0.13
Albumin	Reference	(-)	0.93 (0.86 – 1.02)	0.11	0.93 (0.85 – 1.01)	0.10
CRP	Reference	(-)	1.00 (0.98 – 1.03)	0.75	1.00 (0.97 – 1.02)	0.67
Smoking	Reference	(-)	0.92 (0.45 – 1.86)	0.81	1.59 (0.80 – 3.14)	0.18

Alcohol use	Reference	(-)	1.03 (0.63 – 1.68)	0.92	0.70 (0.42 – 1.16)	0.17
Level of education*						
- Intermediate	Reference	(-)	0.47 (0.27 – 0.79)	0.004	0.87 (0.50 – 1.50)	0.61
- High	Reference	(-)	0.71 (0.41 – 1. 23)	0.22	0.80 (0.44 – 1.46)	0.46

*low education is reference group; Abbreviations: 95% CI: 95% confidence interval; BMI: Body Mass Index; eGFR: estimated glomerular filtration rate; CIS: Checklist Individual Strength; g/kg/d: grams per kilogram per day; CRP: C-reactive protein; OR: Odds Ratio; SBP: systolic blood pressure; Tx: transplantation.

Table S2. Association of protein intake* (per 0.1 g/kg/d increment) with fatigue in KTR

	**No Fatigue**		**Moderate Fatigue**		**Severe Fatigue**	
**CIS**	**<20**		**20-34**		**≥ 35**	
	Odds Ratio (95% CI)	P-value	Odds Ratio (95% CI)	P-value	Odds Ratio (95% CI)	P-value
Crude	Reference	(-)	0.91 (0.85 – 0.98)	0.01	0.85 (0.79 – 0.92)	<0.001
Model 1	Reference	(-)	0.91 (0.85 – 0.98)	0.01	0.85 (0.79 – 0.92)	<0.001
Model 2	Reference	(-)	0.91 (0.84 – 0.98)	0.008	0.84 (0.78 – 0.91)	<0.001
Model 3	Reference	(-)	0.91 (0.85 – 0.99)	0.02	0.85 (0.79 – 0.93)	0.001
Model 4	Reference	(-)	0.91 (0.85 – 0.99)	0.02	0.86 (0.79 – 0.93)	<0.001
Model 5	Reference	(-)	0.93 (0.86 – 1.02)	0.11	0.85 (0.77 – 0.93)	0.001
Model 6	Reference	(-)	0.92 (0.85 – 1.01)	0.09	0.85 (0.77 – 0.94)	0.001

*Protein intake in g/kg/d with adjustment for underweight and obesity: a BMI <20 is adjusted to a BMI of 20 and a BMI ≥ 30 is adjusted to a BMI of 27.5.
Abbreviations: 95% CI: 95% confidence interval; CIS: Checklist Individual Strength; g/kg/d: grams per kilogram per day
Model 1: adjusted for age, sex, BMI
Model 2: adjusted for model 1 variables plus eGFR, proteinuria and primary kidney disease.
Model 3: adjusted for model 2 variables plus time after transplantation, pre-emptive transplantation, living kidney donor, mycophenolic acid use, cyclosporin use and history of combined organ transplantation.
Model 4: adjusted for model 3 variables plus diabetes, systolic blood pressure and cholesterol and statin use.
Model 5: adjusted for model 4 variables plus hemoglobin, ferritin, vitamin B12, albumin, C-reactive protein.
Model 6: adjusted for model 5 variables plus smoking status, alcohol use and level of education.

Table S3. Association of protein intake* (per 0.1 g/kg/d increment) with Quality of Life.

	PCS		MCS	
	**β (95% CI)**	**P-value**	**β (95% CI)**	**P-value**
Crude	0.62 (0.32 - 0.91)	<0.001	0.19 (-0.07 - 0.44)	0.16
Model 1	0.62 (0.34 – 0.90)	<0.001	0.18 (-0.07 - 0.43)	0.16
Model 2	0.63 (0.34 – 0.91)	<0.001	0.24 (-0.01 - 0.50)	0.06
Model 3	0.54 (0.26 - 0.82)	<0.001	0.18 (-0.07 – 0.44)	0.16
Model 4	0.48 (0.19 – 0.76)	0.001	0.17 (-0.09 – 0.43)	0.21
Model 5	0.44 (0.14 – 0.75)	0.004	0.22 (-0.07 – 0.50)	0.13
Model 6	0.44 (0.14 – 0.75)	0.005	0.22 (-0.07 – 0.50)	0.13

*Protein intake in g/kg/d with adjustment for underweight and obesity: a BMI <20 is adjusted to a BMI of 20 and a BMI ≥ 30 is adjusted to a BMI of 27.5.
Abbreviations: 95% CI: 95% confidence interval; g/ d: grams per day; g/kg/day: grams per kilogram per day; MCS: Mental component summary score; PCS: Physical component summary score.
Model 1: adjusted for age, sex, BMI.
Model 2: adjusted for model 1 variables plus eGFR, proteinuria and primary kidney disease.
Model 3: adjusted for model 2 variables plus time after transplantation, pre-emptive transplantation, living kidney donor, mycophenolic acid use, cyclosporin use and a history of combined organ transplantation.
Model 4: adjusted for model 3 variables plus diabetes, systolic blood pressure and cholesterol and statin use.
Model 5: adjusted for model 4 variables plus hemoglobin, ferritin, vitamin B12, albumin, C-reactive protein.
Model 6: adjusted for model 5 variables plus smoking status, alcohol use and level of education.
